# Patient experiences and perceived value of genetic testing in inherited retinal diseases: a cross-sectional survey

**DOI:** 10.1038/s41598-024-56121-2

**Published:** 2024-03-05

**Authors:** Alexis Ceecee Britten-Jones, Joshua Schultz, Heather G. Mack, Lisa S. Kearns, Aamira J. Huq, Jonathan B. Ruddle, David A. Mackey, Alex W. Hewitt, Thomas L. Edwards, Lauren N. Ayton

**Affiliations:** 1 Department of Optometry and Vision Sciences, Faculty of Medicine, Dentistry and Health Sciences, The University of Melbourne, Parkville, VIC 3010 Australia; 2 Centre for Eye Research Australia, Royal Victorian Eye and Ear Hospital, Melbourne, Australia; 3 Department of Surgery (Ophthalmology), Faculty of Medicine, Dentistry and Health Sciences, University of Melbourne, Parkville, Australia; 4 Department of Genomic Medicine, Royal Melbourne Hospital, Parkville, VIC Australia; 5 Department of Medicine, University of Melbourne, Parkville, VIC Australia; 6 Menzies Institute for Medical Research, School of Medicine, University of Tasmania, Hobart, TAS 7000 Australia; 7 Centre for Ophthalmology and Visual Science, Lions Eye Institute, The University of Western Australia, Nedlands, WA 6009 Australia

**Keywords:** Genetics research, Retinal diseases, Genetic testing

## Abstract

This study evaluated patient experiences with genetic testing for inherited retinal diseases (IRDs) and the association between underlying knowledge, testing outcomes, and the perceived value of the results. An online survey was distributed to adults with IRDs and parents/guardians of dependents with IRDs who had had genetic testing. Data included details of genetic testing, pre- and post- test perceptions, Decision Regret Scale, perceived value of results, and knowledge of gene therapy. Of 135 responses (85% from adults with IRDs), genetic testing was primarily conducted at no charge through public hospitals (49%) or in a research setting (30%). Key motivations for genetic testing were to confirm IRD diagnosis and to contribute towards research. Those who had received a genetic diagnosis (odds ratio: 6.71; p < 0.001) and those self-reported to have good knowledge of gene therapy (odds ratio: 2.69; p = 0.018) were more likely to have gained confidence in managing their clinical care. For over 80% of respondents, knowing the causative gene empowered them to learn more about their IRD and explore opportunities regarding clinical trials. Key genetic counselling information needs include resources for family communications, structured information provision, and ongoing genetic support, particularly in the context of emerging ocular therapies, to enhance consistency in information uptake.

## Introduction

Inherited retinal diseases (IRDs) caused by variants in single genes are estimated to occur in about 1 in 2000 to 4000 individuals^1^, and are a major cause of blindness in working-age adults^2,3^. IRDs are clinically and genetically heterogenous; to date, disease causing variants in over 300 genes have been identified^4^. Genetic testing can be used to identify the causative gene. The diagnostic yield of IRD testing using next generation sequencing is currently around 60%^5^, but varies between different phenotypes and patterns of inheritance. Following the U.S. Food and Drug Administration approval of the world first direct-to-human gene replacement therapy for an IRD, voretigene neparvovec-rzyl, in 2017 (and by the Australian Therapeutic Goods Administration in 2020), a number of other gene-specific treatments are under development^6^. Identifying a patient’s causative gene is a fundamental step for determining their suitability for participating in clinical trials for emerging gene-specific treatments and receiving approved treatments. Genetic testing for IRDs is a part of ophthalmology practice standard-of-care^7,8^, but access varies among countries due to differing national policies^9,10^. A confirmed genotype also provides information on disease inheritance and risk to other family members^11^. Additionally, genetic testing can also identify people at risk of additional systemic manifestations (syndromic IRD); for example, those with Usher syndrome that might benefit from hearing testing and hearing interventions.

Several studies have investigated patient attitudes towards IRD genetic testing and found most patients believe this should be offered to all affected individuals^12,13^. However, few studies have evaluated the influence of genetic testing outcomes and patient experiences on post-testing behaviour and subsequent health-care decision-making. A 2022 cross-sectional survey of participants from a Japanese public hospital found that those who received positive results were more likely to have found benefits from testing than those who received a negative/inconclusive result, but both groups found genetic testing to be informative^14^. Qualitative studies have also found that, despite broadly positive views about genetic testing, receiving results is a complex emotional experience^15^ and the associated psychosocial risks are not well defined^16^.

The aim of this study was to evaluate participants’ motivations and experiences with IRD genetic testing and the association between patients’ knowledge and experiences on the perceived value of the genetic test results.

## Results

### Participant demographics and genetic testing outcomes

Between 1st November 2021 and 21st March 2023, 148 survey responses were received (response rate 65%), including 124 from adults with IRDs (84%) and 24 from parents/guardians of children/dependents with IRDs (16%) living in Australia. Only fully complete responses (n = 135) were included in the analysis.

**Figure 1:**
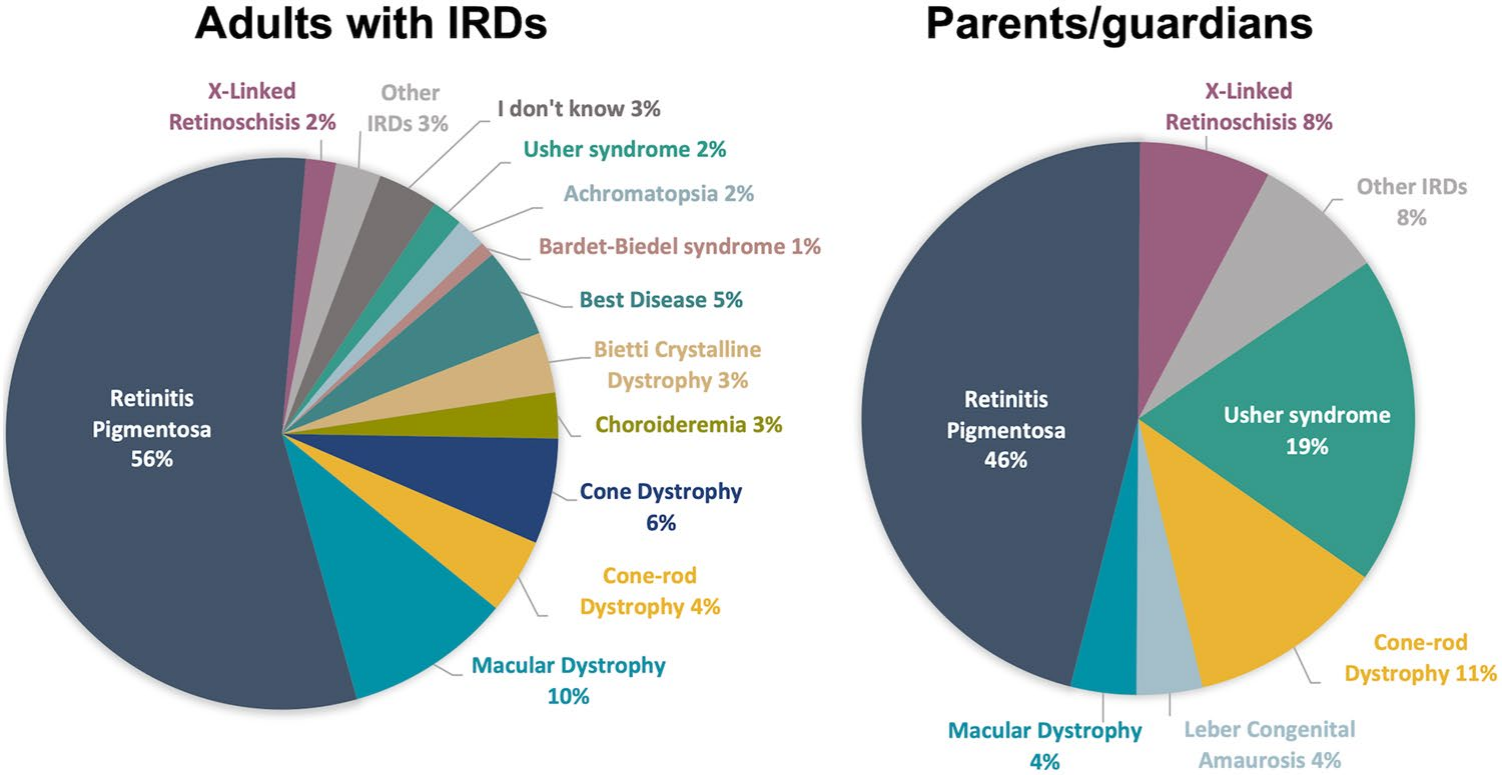
Self-reported inherited retinal disease (IRD) diagnoses of survey respondents (total n = 135). Self-reported diagnoses of (**A**) Adults with IRDs (n = 113) and (**B**) Parents/guardians for their dependents with IRDs (n = 22).

Respondents’ demographics are shown in Table 1. The mean age of all respondents was 48 years, and 54% were female. The more common IRDs were retinitis pigmentosa (56%), macular dystrophies (10%), and cone dystrophy (6%) in adult participants (Fig. 1). For parents/guardians, the most common IRDs amongst their dependents were retinitis pigmentosa (46%) and Usher syndrome (19%). Most respondents recalled genetic testing through public hospitals or research programs, both of which provide testing at no cost to the patient.

**Table 1:** Participant demographics.

	Adult with an IRD (n = 113)	Parent/guardian of a dependent with an IRD (n = 22)
Age, mean ± SD	48 ± 17	47 ± 8
Gender, n (%) male	57 (50%)	3 (13.6%)
Highest level of education, n (%)		
Trade certificate	16 (14.2%)	6 (27.3%)
Bachelor degree	39 (34.5%)	5 (22.7%)
Postgraduate degree	26 (23.0%)	7 (31.8%)
Other educational level/prefer not to say	32 (28.3%)	4 (18.2%)
Genetic testing program, n (%)		
Australian public genetics clinic	53 (46.9%)	13 (59.0%)
Australian research program	38 (33.6%)	3 (13.6%)
Australian private clinical geneticist	7 (6.2%)	2 (9.1%)
Overseas	6 (5.3%)	4 (18.2%)
Other, or do not remember	9 (8.0%)	0 (0%)
Time since genetic testing, n (%)		
< 6 months	51 (45.1%)	3 (13.6%)
6 months to 2 years	29 (25.7%)	9 (40.9%)
2–5 years	15 (13.3%)	5 (22.7%)
Over 5 years	18 (15.9%)	5 (22.7%)

Among all respondents, 44% had another family member who also has an IRD, most commonly the affected individual’s sibling (30% of all respondents), parent (13%), grandparents (10%), or aunt/uncle (10%). Prior to their most recent genetic test, 77% had never had a genetic test before for any condition. However, 35% of respondents had other family members that had had genetic testing, primarily for diagnosis of an ocular condition (70%).

From genetic testing, 73% of respondents reported receiving a positive finding about the gene(s) associated with their or their child/dependent’s IRD. Of those who did, 62% could still recall their genetic diagnosis, with no significant differences between those who had genetic testing through public hospitals and research (p = 0.05) or other settings (p = 0.59). The remaining respondents either could not remember (23%), preferred not to say (4%), or only recalled their clinical diagnosis (e.g., X-linked retinitis pigmentosa).

Of the 99 respondents who received a conclusive diagnostic finding, 55% of adults and 45% of parents/guardians felt confident that they have a clear understanding of what their genetic test results mean; 34% of adults and 36% of parents/guardians felt like they understood somewhat, and 11% of adults and 18% of parent/guardians felt like they had no or little understanding of what the genetic results mean.

**Figure 2:**
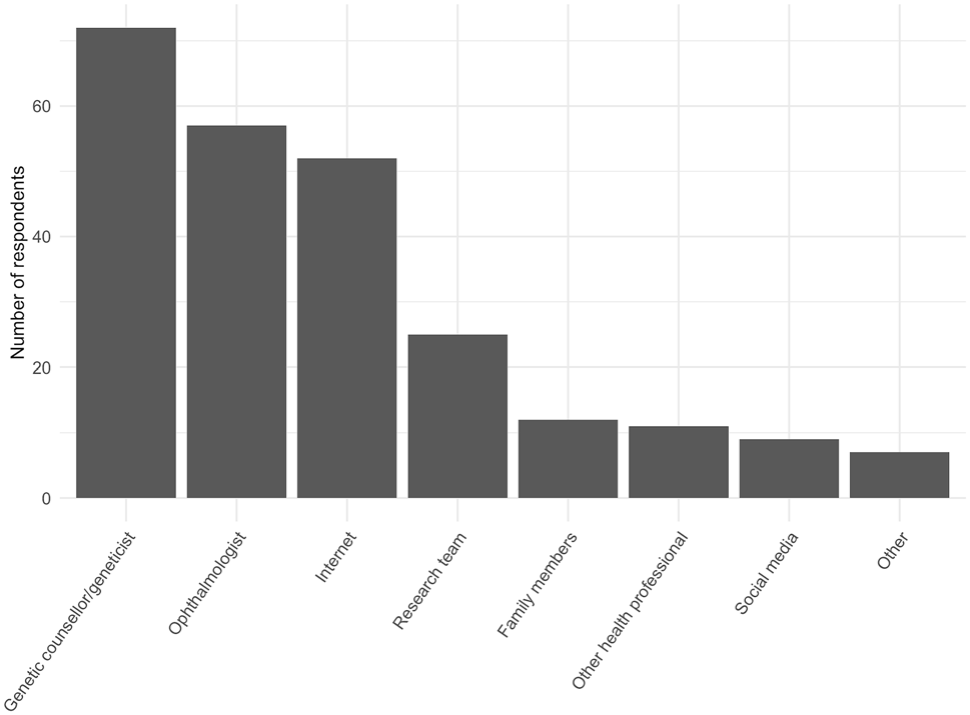
Participants’ primary sources of educational information after receiving genetic test results.

The main sources of information for respondents after receiving their genetic testing results were genetic counsellors/geneticists (53%) and ophthalmologists (42%). Other key sources were the internet (39%) and research teams (19%; Fig. 2).

### Pre-test expectations and motivations

**Figure 3:**
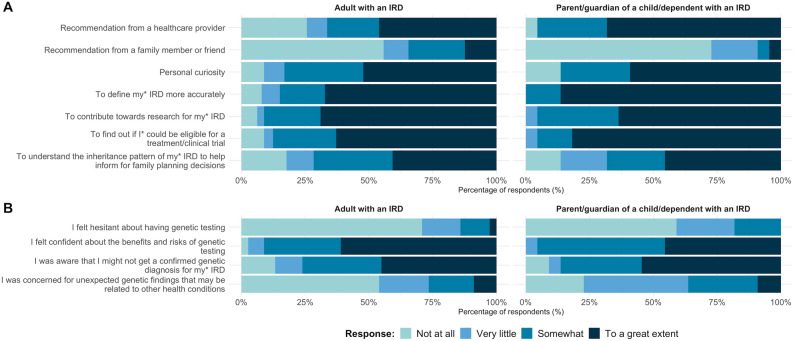
Factors that influence participants’ decision to have inherited retinal disease genetic testing and participants’ pre-test perceptions. (**A**) Degree in which different factors influenced participants’ decision to have genetic testing. (**B**) Level of concern before the genetic test.

Figure 3 shows the degree to which different factors influenced participants’ decision to have genetic testing and their perception of the potential outcomes prior to getting genetic testing.

For adults with IRDs, key factors that greatly influenced their decisions to have genetic testing were to contribute towards research (69% indicated to a great extent), to define their IRD more accurately (67%), and to find out if they could be eligible for a treatment/clinical trial (63%). For parents/guardians, the key factors were to define their child’s IRD more accurately (86%) and to find out about their eligibility for treatments/clinical trials (82%). In addition, recommendations from a health-care provider (68%) played a bigger role in influencing parents/guardians’ decision to have genetic testing for their dependents than it did for adults with IRDs (46%).

Most respondents indicated feeling no or very little hesitation about having genetic testing (85%). However, only 61% of adults and 45% of parents/guardians felt confident that they knew the benefits and risks of genetic testing beforehand, and 45% of adults and 55% of parents/guardians knew that the test might not obtain a confirmed diagnosis for their or their child/dependent’s IRD. Before testing, 72% of respondents had no or very little concern of unexpected genetic findings that may be related to other health conditions.

### Post-test attitudes and perceived value of ocular genetic testing results

Most respondents (94% adults and 82% parents/guardians) had discussed their genetic testing results with another family member. Of those who did, 83% reported that they were very or somewhat confident about explaining these results to their family member, while 17% had little or no confidence. Reasons for not discussing their genetic testing results with family members included not thinking that they would understand or not wanting to scare them, and for parents/guardians, not thinking that the results were relevant to other family members.

The overall mean score for the decision regret scale was 6 (range 0–60) out of 100, with 65% of respondents scoring 0, indicating that most people had no regret towards genetic testing. There was no significant difference between mean scores of respondents who did and who did not get a conclusive diagnosis from genetic testing (5.3 vs 5.8 out of 100; p = 0.78) or between affected adults and parents/guardians (5.7 vs 5.7 out of 100; p > 0.99).

**Figure 4:**
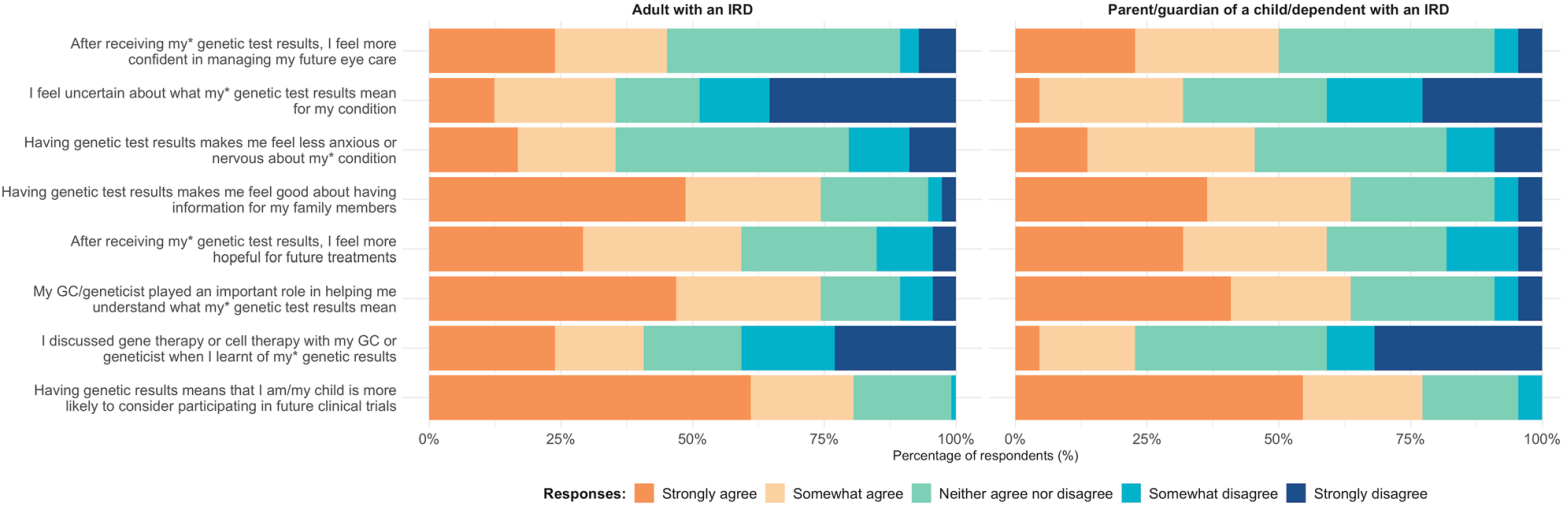
Adults with inherited retinal diseases and parents’/guardians’ level of agreement with perceived value of genetic testing statements after receiving test outcomes.

Figure 4 shows respondents’ perceptions on the perceived value of genetic testing results. The distribution of responses between adults with IRDs and parents/guardians were similar.

After having genetic testing, 35% of all respondents reported that they still felt uncertain about what the genetic test results meant for their or their child/dependent’s IRD. For over half the respondents, having genetic test results did not influence their confidence in managing their or their dependent’s future eye care (54%) nor did it make them less anxious or nervous about their IRD (63%). However, 73% of respondents agreed that having genetic test results made them feel good about having information for other family members. For 60% of respondents, having genetic test results made them more hopeful about future treatments, and almost 80% agreed that genetic testing results means that they are more likely to consider participating in future clinical trials.

Most respondents (73%) indicated that their genetic counsellor or geneticist played a big role in helping them understand what their or their child/dependent’s genetic test results mean. However, only 38% recalled having discussed gene or cell therapies with their geneticist/counsellor when they had their genetic test results disclosed.

### Knowledge of ocular gene therapy

Although only 39% of participants self-reported having good knowledge about gene therapy for IRDs, more than twice as many (83%) would consider participating in a gene therapy clinical trial or treatment if it was available to them or their child/dependent right now.

Regarding knowledge of gene therapy methods, 66% knew that gene and stem cell therapies are not the same treatment, but only 37% correctly indicated that gene therapy for the eye is not suitable for all stage of disease (Supplemental Fig. S1). For awareness of treatment outcomes, 70% knew that having gene therapy does not mean that a person will not pass their IRD to any children they may have in the future; however, only 48% knew that the main goal of gene therapy is to slow down the disease, and 40% knew that treatment complications, such as permanent blindness, are possible with an approved gene therapy treatment.

### Association between genetic testing outcomes and behaviour

Univariate and multivariate logistic regression were used to evaluate whether participant characteristics, knowledge, and genetic testing outcomes influenced participants’ perception of their care (Supplemental Table S1). None of the demographic variables were related to whether genetic testing results made participants more confident regarding their eye care management. However, those who had received a positive/conclusive diagnostic result from their genetic test were more likely to gain confidence in managing their/their dependent’s eye care after having genetic testing (OR: 6.71 [95% CI 2.45–21.9]). Participants who self-reported to have good knowledge of gene therapy were about three times as likely (OR: 2.69 [95% CI 1.19–6.20]) to feel more confident in managing their/their dependent’s eye care following genetic testing.

## Discussion

This study demonstrates the important value of IRD genetic testing for affected families, regardless of whether the results directly affect their current clinical care or management. Most adults with IRDs and parents/guardians felt no regret about their decision to have genetic testing. Furthermore, knowing the genetic cause of their IRDs empowers patients to know about their condition and increased their desire to participate in future research and clinical trials.

In the present study, the leading motivating factors for undergoing genetic testing were to contribute towards research and to have an IRD defined more accurately, followed by the desire to determine eligibility for a treatment/clinical trial. These findings differ from previous studies where qualification or information for clinical trials were the leading motivating factor for IRD genetic testing^14,15^. The difference might be because we recruited from both public hospital and research clinic settings. The findings support previous studies in highlighting the importance of knowledge and decision control in empowering patients to learn about the cause of their IRD^17^.

Most participants viewed the benefits of diagnostic genetic testing to outweigh its potential risks, showing generally positive decisional balance, consistent with findings from qualitative studies^15,16^ and in other rare diseases in the UK 100,000 genome project^18^. However, only about 40% of respondents found having genetic testing results provided a sense of relief from uncertainty or gave them more confidence in managing their future eye care. Participants who received a positive diagnostic finding, and those with good self-reported knowledge of gene therapy, were more likely to gain confidence in managing their future eye care after receiving genetic test results. These findings support the notion, as it has been reported in other rare diseases, that the sense of empowerment associated with genetic testing can be enhanced by improved health literacy and shared decision-making^18–20^.

Most respondents (88%) had discussed their genetic testing results with another family member, and most (84%) knew whether someone else in the family had also had genetic testing. This supports findings from a previous study in Japan that also found people with IRDs generally share their genetic results with other family members^14^. Understanding family history is important for informing patterns of IRD inheritance and aids in identifying the genetic diagnosis^21^. Conversely, knowing the causative gene in an affected individual can guide the testing of other family members to identify those at risk of inheriting the condition or carrier status^8,11^. While the majority of participants felt comfortable discussing genetic results with family members, possibly indicating a growing educational trend, 1 in 6 of respondents reported a lack of confidence in engaging in these discussions, highlighting a need for additional support in this domain. Likewise, qualitative studies have found that patients with IRDs reported generally good experiences with informing relatives about IRD-related test results^22^, but wanted more resources on how to share the information^23^.

Communication of genetic test results to other family members can be challenging and complicate family dynamics^24^. A previous study of public hospital patients in Japan found no difference in the desire to share between those who did and did not receive a positive result in sharing results with their family^14^. In hereditary cancer, patient opinions on intra-familial disclosure of their genetic testing results were divided. Family communication was found to be influenced by not only the nature of the relationship, but also by patients’ understanding of the importance and perceived relevance of this information to other family members, as well as their anticipated reactions^24,25^. Targeted post-test genetic counselling information regarding relevance to other family members is likely valuable to patients with IRDs^26^.

Regarding genetic counselling information uptake, at least 1 in 5 participants did not recall knowing before their genetic test that that they might not receive a confirmed diagnosis, and a third of respondents still felt uncertain about what the genetic test results meant for their or their child/dependent’s IRD. Similarly, only 1 in 3 respondents recalled discussing gene or cell therapies when they had their genetic test results disclosed, indicating a need for better counselling regarding possible therapies. As such, development of guidelines and resources to support IRD genetic counselling could encourage consistency and coherence in this process. Providing written information, such as leaflets and online sources, and resources that accommodate for accessibility needs, such as low vision, hearing impairments, and intellectual disabilities, could enhance the uptake of genetic counselling information.

Genetic testing entails not only ordering the test and interpreting the results in the context of clinical findings, but also comprehensive patient counselling^8,11^, including pre-test counselling to help patients establish realistic expectations and prepare for the implications of the results^15,16^. Furthermore, receiving a genetic diagnosis does not mitigate emotions like uncertainty and anxiety. The Clinical Genome Resource has developed general guidance to aid clinicians in discussing genetic testing consent with patients and disclosing the results^27^. Pre-test counselling should include why a genetic test is being offered, the scope and goal of genetic testing, possible outcomes, secondary and incidental findings, what the test can and cannot determine, a brief overview of how results may affect management, and logistics including insurance issues^28^. Additional considerations that are specific to IRDs include the likelihood of a positive diagnostic result (currently around 60% with next generation sequencing panels)^5^, association with syndromic findings, and implications for emerging gene and cell therapy treatments^6,29^.

Within the last five years, genetic testing for IRDs has become more accessible within mainstream medicine^30,31^ and through research programs^10,32–34^, and gene and cell therapies have been developed^6^. With improved access to diagnostic genetic testing, it is important to evaluate and enhance the genomics literacy of eye care professionals to manage expectations and meet their information needs^36^. Diversity in genetic counselling experiences may be more apparent in regions with inequitable access to genetic counsellors and genetic services^37^. Our findings emphasise the important role played by genetic counsellors, geneticists, and other professionals with genetic expertise in helping patients understand their genetic testing results. Establishing systems for effective communications and results-sharing between genetic providers (e.g., genetic counsellors, geneticists) and eye care providers (e.g., ophthalmologists, optometrists) is crucial to keep all care providers informed about the patient’s clinical care plan. Increased support for multi-disciplinary frameworks will enhance the clinical value of the genetic testing and improve the integrated care of IRDs.

This study captured perspectives from individuals who underwent genetic testing across various settings, reflecting a broad range of experiences. Both the public hospital and research program we recruited from offered pre- and post-test genetic counselling; however, the depth of discussion may have led to variations in participants' initial understanding levels. Most responses were from people who had genetic testing through no cost programs. Many participants had their testing through industry-sponsored genetic testing programs, specifically aimed at diagnosing rod-cone dystrophies. This has influenced the representation of IRDs in our study cohort, leading to a smaller proportion of macular dystrophies than we would otherwise expect. We did not investigate monetary values associated with those who may have paid for testing. Genetic testing through private providers is less common in Australia^35,38^; those who have paid for testing may have different counselling experiences and perceived value of results, particularly of negative findings. The impact of payment on patient experiences with genetic testing is a potential area for future research.

Genetic testing results were self-reported, and we were unable to validate how many correctly reported their gene to assess the accuracy of their knowledge and the understanding of their results. Many of our respondents were highly educated, with 27% having a postgraduate degree. The self-enrolment method of recruitment meant that findings are potentially biased towards people interested in research, who might be more inclined to acquire information. We did not consider the perspectives of individuals who had decided not to have genetic testing. Considerations for future work include investigating the perceptions of these patients and how this impacts their health behaviour, to assist in the development of a comprehensive framework on the provision of genetic testing and counselling for IRDs.

## Conclusions

Genetic testing can empower patients to learn about the cause of their IRDs and their eligibility for upcoming clinical trials and approved treatments. This is particularly important in the current era of personalised genomic health and the emergence of IRD gene and cell therapies.

Our study has shown that both the outcome (positive/negative results) and an individual’s self-reported genomic literacy can influence how they respond to their genetic testing results. These factors should be considered when counselling patients on their IRD genetic test results.

Resources to facilitate family communication and encourage consistency and coherence in IRD genetic counselling could improve information uptake for patients. The time of genetic testing is often very emotionally charged; hence, structured information provision can help patients to absorb and retain the most relevant information to them. These resources would also empower patients to take a stronger role in their healthcare, enabling a shared decision-making process between patients and their clinicians.

Finally, an important finding of this study was that there was very little regret from the participants from having engaged in genetic testing, no matter what their clinical outcome was. This brings assurance to clinicians advocating for these tests, especially admist the ongoing emphasis on identifying the genetic cause of all IRDs to ensure readiness for emerging therapies.

## Methods

The project was approved by the University of Melbourne Human Research Ethics Committee (Reference: 2021-22502-23169-4) and was performed in accordance with the principles of the Declarations of Helsinki. All participants provided informed consent to participate. Eligible participants were either adults who had undergone IRD genetic testing or parents/guardians of children/dependents who had had IRD genetic testing, who are living in Australia. To participate, participants must have had their genetic testing results already disclosed to them.

### Survey design and assessment

An anonymous, online, self-administered survey was prepared based on patient-reported experiences from published studies^16,26^ and in consultation with a multidisciplinary ocular genetics team (ophthalmologists, IRD researchers, academic optometrists, genetic counsellors, and a clinical geneticist). The draft survey was individually assessed by five adults, or parent/guardians of dependents, with IRDs, who provided feedback on the clarity of the questions, flow, and accessibility.

This survey comprised six sections:

1. Demographics and details of the genetic testing (self-reported)
2. Pre-test expectations and motivations to have genetic testing
3. Outcomes of genetic testing and level of understanding of results
4. Regret towards having genetic testing
5. Perceived value of ocular genetic testing results
6. Knowledge of ocular gene therapy

Regret towards genetic testing was assessed using the validated Decision Regret Scale, which has been shown to correlate with satisfaction in health-care decisions and quality of life^39^. The Decision Regret Scale was used to measure the level of distress or remorse surrounding the decision to undertake genetic testing. Scores were quantified to a 0–100 scale according to validated protocols, where a score of 0 means no regret, and a score of 100 means high regret.

For assessing knowledge of ocular gene therapy, participants were first asked to grade their own knowledge on this topic (perceived knowledge), then their knowledge was assessed using questions adapted from the Attitudes to Gene Therapy-Eye (AGE-Eye) Tool^29,40,41^. Five questions were selected to assess knowledge of gene therapy. Item responses were collapsed to a three-point scale (agree, disagree, neither agree nor disagree) to assess knowledge.

### Data collection

An invitation to participate in the anonymous survey was sent to Australia-based participants in the Victorian Evolution of IRDs Natural History Registry^10^ who had had genetic testing and who had consented to being contacted about future research, as well as patients who had had genetic testing and results disclosed with the Royal Victorian Eye and Ear Hospital Ocular Genetics Clinic during the study period. Both avenues included pre- and post-test genetic counselling. A total of 226 invitations to participate were sent between 1 November 2021 and 21st March 2023. The study was also advertised through national patient-support group newsletters (Retina Australia, Vision Australia).

### Data analysis

Sample size was estimated using an IRD population prevalence of 1 in 2000^42^, and a genetic testing prevalence of 10% in Australia^35^. Based on 95% confidence level and 5% margin of error, the estimated sample size was 135 participants.

Statistical analysis was performed using R for statistical consulting (v4.1.2; R Core Team 2021).

Only fully complete responses were included in the analysis.

Descriptive methods were used to summarise the frequency and percentages of responses for categorical measures. Normality testing was performed using Shapiro Wilks test and, based on the data distribution, continuous variables were summarised as median (SD) for normally-distributed variables or median (IQR) for skewed variables.

For continuous variables, responses were compared between respondent types (adults with IRD vs parent/caregiver) and genetic testing outcomes (positive/negative diagnosis from genetic test) using a two-sample t-test for normally distributed variables and Wilcoxon rank-sum test for skewed variables. Fisher’s exact test was used to compare data relating to the proportions of respondents across different groups.

Univariate and multivariate binocular logistic regression analyses were performed to assess factors related to whether having genetic testing results made participants more confident towards managing their eye care. Predictors included: respondent type, age, gender, education, family history, genetic testing program, time since genetic test, genetic testing history, level of hesitation about genetic testing, awareness of outcomes, outcome of diagnosis, whether gene therapy was discussed during genetic counselling, willingness to participate in a clinical trial, perceived and actual knowledge of gene therapy, and regret towards genetic testing. Variables significantly associated with the outcome were included in the multivariable model. A p-value of < 0.05 was considered statistically significant.

### Supplementary Information

Supplementary Information.

## Data Availability

Non-identifiable data supporting the findings of this study are available from the corresponding author (ACBJ) upon reasonable request for ethically approved projects.

## References

[1] Hanany, M., Shalom, S., Ben-Yosef, T. & Sharon, D. Comparison of worldwide disease prevalence and genetic prevalence of inherited retinal diseases and variant interpretation considerations. *Cold Spring Harb. Perspect. Med.* **14** (2024). 10.1101/cshperspect.a041277 PMC10835612 37460155

[2] Heath Jeffery, R. C. *et al.* Inherited retinal diseases are the most common cause of blindness in the working-age population in Australia. *Ophthalmic Genet.* **42**, 431–439 (2021). 10.1080/13816810.2021.1913610 PMC8315212 33939573

[3] Liew G, Michaelides M, Bunce C (2014). A comparison of the causes of blindness certifications in England and Wales in working age adults (16–64 years), 1999–2000 with 2009–2010. BMJ Open.

[4] SP Daiger, BJF Rossiter, J Greenberg, A Christoffels & Hide., W. *Data services and software for identifying genes and mutations causing retinal degeneration.* https://sph.uth.edu/RetNet/ (1998).

[5] Britten-Jones AC (2022). The diagnostic yield of next generation sequencing in inherited retinal diseases: A systematic review and meta-analysis. Am. J. Ophthalmol..

[6] Britten-Jones AC (2021). The safety and efficacy of gene therapy treatment for monogenic retinal and optic nerve diseases: A systematic review. Genet. Med..

[7] The Royal Australian and New Zealand College of Ophthalmologists: Guidelines for the assessment and management of patients with inherited retinal diseases (IRD). (2020). Accessed 1 April 2023.

[8] Stone EM (2012). Recommendations for genetic testing of inherited eye diseases: Report of the American Academy of Ophthalmology task force on genetic testing. Ophthalmology.

[9] Bottazzi L (2023). Understanding the propensity to undergo genetic testing in patients affected by inherited retinal diseases: A twelve-item questionnaire. Ophthalmic Genet..

[10] Britten-Jones AC, O'Hare F, Edwards TL, Ayton LN (2022). Victorian evolution of inherited retinal diseases natural history registry (VENTURE study): Rationale, methodology, and initial participant characteristics. Clin. Exp. Ophthalmol..

[11] Lam BL (2021). Genetic testing and diagnosis of inherited retinal diseases. Orphanet J. Rare Dis..

[12] Bong C, Potrata B, Hewison J, McKibbin M (2010). Attitudes of patients and relatives/carers towards genetic testing for inherited retinal disease. Eye.

[13] Willis TA (2013). Understanding of and attitudes to genetic testing for inherited retinal disease: A patient perspective. Br. J. Ophthalmol..

[14] Inaba A (2022). Perception of genetic testing among patients with inherited retinal disease: Benefits and challenges in a Japanese population. J. Genet. Couns..

[15] Krauss E (2022). Experiences of genetic testing among individuals with retinitis pigmentosa. Ophthalmic. Genet..

[16] Combs R (2013). Understanding the impact of genetic testing for inherited retinal dystrophy. Eur. J. Hum. Genet..

[17] McAllister M, Dunn G, Todd C (2011). Empowerment: Qualitative underpinning of a new clinical genetics-specific patient-reported outcome. Eur. J. Hum. Genet..

[18] Peter M (2022). Participant experiences of genome sequencing for rare diseases in the 100,000 Genomes Project: A mixed methods study. Eur. J. Hum. Genet..

[19] Yuen J (2020). Evaluating empowerment in genetic counseling using patient-reported outcomes. Clin. Genet..

[20] De Santis, M., Hervas, C., Weinman, A., Bosi, G. & Bottarelli, V. Patient empowerment of people living with rare diseases. Its contribution to sustainable and resilient healthcare systems. *Ann. Ist. Super. Sanita* **55**, 283–291 (2019). 10.4415/ANN_19_03_15 31553324

[21] Méjécase C (2020). Practical guide to genetic screening for inherited eye diseases. Ther. Adv. Ophthalmol..

[22] Saelaert M (2021). A qualitative study among patients with an inherited retinal disease on the meaning of genomic unsolicited findings. Sci. Rep..

[23] McKibbin M (2014). Current understanding of genetics and genetic testing and information needs and preferences of adults with inherited retinal disease. Eur. J. Hum. Genet..

[24] Young AL (2019). Challenges and strategies proposed by genetic health professionals to assist with family communication. Eur. J. Hum. Genet..

[25] Chivers Seymour, K., Addington-Hall, J., Lucassen, A. M. & Foster, C. L. What facilitates or impedes family communication following genetic testing for cancer risk? A systematic review and meta-synthesis of primary qualitative research. *J. Genet. Couns* **19**, 330–342 (2010). 10.1007/s10897-010-9296-y 20379768

[26] Lemke AA (2021). Patient-reported outcomes and experiences with population genetic testing offered through a primary care network. Genet. Test. Mol. Biomarkers.

[27] Ormond KE (2019). Developing a conceptual, reproducible, rubric-based approach to consent and result disclosure for genetic testing by clinicians with minimal genetics background. Genet. Med..

[28] Hallquist MLG (2021). Application of a framework to guide genetic testing communication across clinical indications. Genome. Med..

[29] Mack, H. G. *et al.* Survey of perspectives of people with inherited retinal diseases on ocular gene therapy in Australia. *Gene Ther.* (2022). 10.1038/s41434-022-00364-z PMC10113139 36183012

[30] Black GC, MacEwen C, Lotery AJ (2020). The integration of genomics into clinical ophthalmic services in the UK. Eye.

[31] McClard CK, Pollalis D, Jamshidi F, Kingsley R, Lee SY (2022). Utility of no-charge panel genetic testing for inherited retinal diseases in a real-world clinical setting. J. Vitreoretin. Dis..

[32] Mansfield, B. C., Yerxa, B. R. & Branham, K. H. Implementation of a registry and open access genetic testing program for inherited retinal diseases within a non-profit foundation. *Am. J. Med. Genet. C Semin. Med. Genet.* **184**, 838–845 (2020). 10.1002/ajmg.c.31825 PMC7540299 32783387

[33] Lidder A, Modi Y, Dedania VS, Brodie SE (2021). DNA testing for inherited retinal disease (IRD): Initial experience with the SPARK/Invitae ‘ID your IRD’ genetic testing panel. Invest. Ophthalmol. Vis. Sci..

[34] Zhao PY, Branham K, Schlegel D, Fahim AT, Jayasundera KT (2021). Association of no-cost genetic testing program implementation and patient characteristics with access to genetic testing for inherited retinal degenerations. JAMA Ophthalmol.

[35] Gocuk SA, Britten-Jones AC, Kerr NM, Lim L, Skalicky S, Stawell R, Ayton LN, Mack HG (2022). Genetic testing of inherited retinal disease in australian private tertiary ophthalmology practice. Clin. Ophthalmol..

[36] Britten-Jones AC (2023). Genetic testing and gene therapy in retinal diseases: Knowledge and perceptions of optometrists in Australia and New Zealand. Clin. Genet..

[37] Cordier C, Lambert D, Voelckel MA, Hosterey-Ugander U, Skirton H (2012). A profile of the genetic counsellor and genetic nurse profession in European countries. J. Community. Genet..

[38] De Roach JN (2013). Establishment and evolution of the Australian inherited retinal disease register and DNA bank. Clin. Exp. Ophthalmol..

[39] Brehaut JC (2003). Validation of a decision regret scale. Med. Decis. Making.

[40] McGuinness MB (2022). Measurement properties of the attitudes to gene therapy for the eye (AGT-Eye) instrument for people with inherited retinal diseases. Transl. Vis. Sci. Technol..

[41] Mack HG (2021). Perspectives of people with inherited retinal diseases on ocular gene therapy in Australia: Protocol for a national survey. BMJ Open.

[42] De Roach J (2020). The Australian inherited retinal disease registry and DNA bank. Tasman. Med. J..

