# Supplementary material for: Patient experiences and perceived value of genetic testing in inherited retinal diseases: a cross-sectional survey
Source: Sci Rep. 2024 Mar 5;14:5403. doi: 10.1038/s41598-024-56121-2 (PMC10914714; doi:10.1038/s41598-024-56121-2)
Supplement: Supplementary file 1 — Supplementary Information. [file 41598_2024_56121_MOESM1_ESM.pdf]

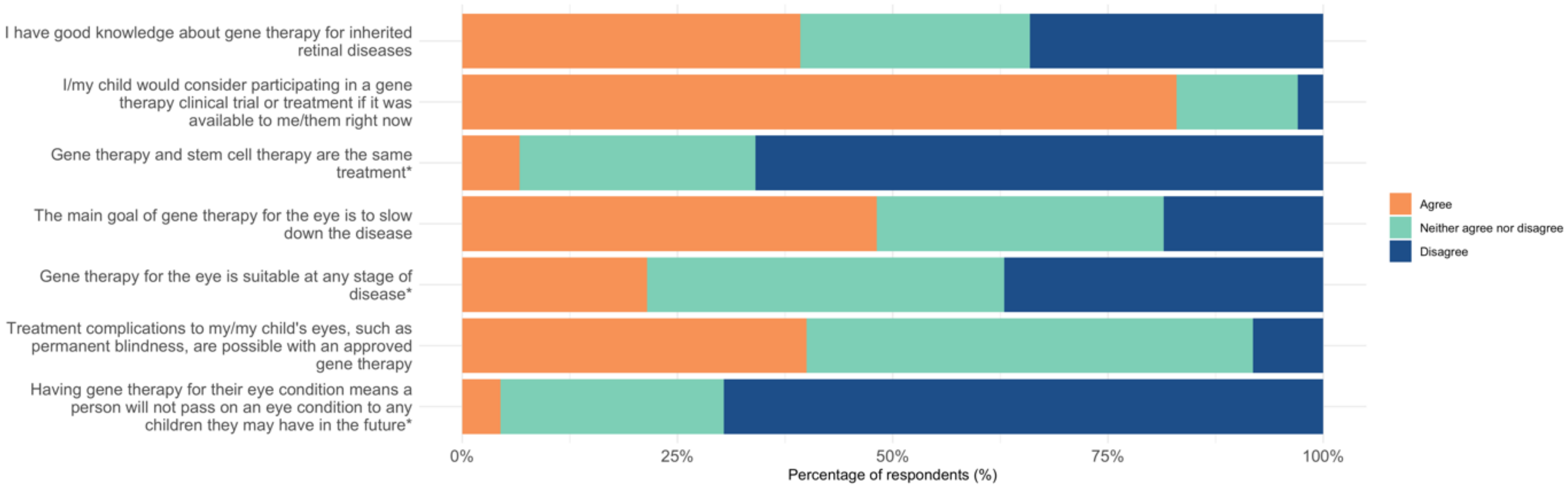

Supplemental Figure 1. Level of knowledge towards gene therapy. Questions include both self-reported knowledge and actual knowledge on the method of treatment and awareness of outcomes. For questions marked with an asterisk (*), “Disagree” was considered to be the correct answer. For all other questions, “Agree” was considered to be correct answer.

Supplemental Table S1: Factors associated with whether having genetic test results made respondents feel more confident in managing their or their child/dependent's eye care.

Factor	Univariate analysis		Multivariate analysis	
	OR (95% CI)	p-value	OR (95% CI)	p-value
**Respondent type**				
Adults with IRDs	Ref		-	
Parents/guardians	1.22 (0.48-3.06)	0.68		
**Age,** years	1.00 (0.98-1.02)	0.77		
**Gender**				
Female or non-binary	Ref		-	
Male	1.71(0.87-3.43)	0.12		
**Highest level of education**				
Secondary school and below, or other	Ref			
Trade certificate	3.00 (1.01-9.59)	0.054		
Bachelor degree	1.17 (0.48-2.86)	0.73	-	
Postgraduate degree	0.80 (0.30-2.11)	0.65		
**Other affected family member**				
No/Not sure	Ref		-	
Yes	1.61 (0.81-3.21)	0.18		
**Genetic testing program**				
Other programs	Ref			
Public clinic	2.30 (0.96-5.78)	0.066	-	
Australian Research program	2.56 (0.98-6.99)	0.060		
**Time since most recent genetic test**				
<6 months	Ref		-	
6 months to 2 years				
>2 years				
**Previous genetic testing for any condition**				
No	Ref		-	
Yes	1.86 (0.85-4.18)	0.13		
**Level of hesitation about genetic testing**				
No/very little	Ref		-	
Somewhat/to a great extent	0.45 (0.15-1.20)	0.13		
**Aware that might not receive a result**				
No/very little	Ref		-	
Somewhat/to a great extent	0.81 (0.36-1.85)	0.61		
**Received positive diagnostic result from genetic test**				
No	Ref		Ref	
Yes	**8.41 (3.25-26.3)**	**<0.001**	**6.71 (2.45-21.9)**	**<0.001**
**Discussed gene therapy with geneticist**				
No	Ref		Ref	
Yes	**3.03 (1.49-6.33)**	**0.003**	1.66 (0.72-3.80)	0.23
**Would participate in a clinical trial if it was available**				
No	Ref		-	
Yes	2.21 (0.87-6.12)	0.11		
**Self-reported to have good knowledge of gene therapy**				
No	Ref		Ref	
Yes	**3.96 (1.93-8.38)**	**<0.001**	**2.69 (1.19-6.20)**	**0.018**
**Assessed knowledge of methods and outcomes of gene therapy**[1]				
Not knowledgeable	Ref		-	
Somewhat knowledgeable	3.33 (0.97-14.3)	0.066		
Knowledgeable	2.56 (0.75-10.0)	0.15		
**Regret towards decision to have genetic testing**				
Any regret (score >0 on DRS)	Ref		Ref	
No regret at all (scored 0 on DRS)	**2.12 (1.03-4.50)**	**0.045**	2.07 (0.90-4.86)	0.088

Statistically significant predictors are in bold. **Abbreviations**: DRS = Decision regret scale. OR = Odds Ratio. CI = Confidence Interval.
[1]Participants who correctly answered all five knowledge questions were considered knowledgeable; those who corrected answered three or four questions were considered somewhat knowledgeable.

Britten-Jones et al. Patient experiences and perceived value of genetic testing in inherited retinal diseases: A cross-sectional survey.
